# How Has the Disappearance of Influenza B/Yamagata Altered the Proportion of Influenza A and B Cases? Early Findings From Post‐COVID Pandemic Global Surveillance Data

**DOI:** 10.1111/irv.70138

**Published:** 2025-07-07

**Authors:** Marco Del Riccio, Jojanneke van Summeren, Saverio Caini, Koos van der Velden, Aura Timen

**Affiliations:** ^1^ Department of Health Sciences University of Florence Florence Italy; ^2^ Nivel Utrecht The Netherlands; ^3^ Department of Primary and Community Care Radboud University Medical Centre Nijmegen The Netherlands

**Keywords:** COVID‐19 pandemic, global surveillance, influenza circulation, influenza epidemiology, influenza prevention, influenza vaccination strategies

## Abstract

We studied worldwide influenza surveillance data (2022–2024), particularly in 145 countries and 260 country‐seasons: influenza A represented 77.2% of identified cases, rising from 72.5% prepandemic, with A‐dominated seasons increasing from 84.6% to 92.3%. During the same period, B/Yamagata was not detected and uncharacterized B cases dropped from 21.0% to 7.5%, possibly reflecting improved surveillance efforts. These results highlight postpandemic changes in influenza circulation and have important implications for vaccine composition, virus monitoring, and global prevention strategies.

## 1 Background

Influenza viruses frequently undergo genetic modifications that can lead to alterations in subtypes and subtype predominance, and this might affect disease burden and vaccination strategies. Before the COVID‐19 pandemic, influenza vaccines contained both influenza B lineages (Victoria and Yamagata) because they had been cocirculating since 1983 [1]. However, from April 2020 onward, the influenza B/Yamagata lineage has not been consistently detected in global surveillance systems, and this disappearance raised questions about its impact on seasonal influenza epidemiology and vaccine composition [2, 3, 4]. In fact, the loss of B/Yamagata might alter the dynamics of influenza A and B cases and potentially impact immunity and seasonal epidemics. The aim of this manuscript is to leverage up‐to‐date surveillance data (through December 2024) from the WHO FluNet database to examine potential shifts in influenza A and B circulation. This study has significant public health implications, as it could timely inform surveillance and vaccine composition decisions and highlight the importance of rapid monitoring in the post‐COVID‐19 era.

## 2 Analysis of Global Influenza Surveillance Data

We analyzed global influenza weekly virological sentinel surveillance data obtained from the WHO FluNet [5] database, covering the period from January 1, 2022, to December 31, 2024. FluNet is a comprehensive web‐based system established by the WHO to support influenza virological surveillance, where National Influenza Centres and other national influenza reference laboratories within the WHO Global Influenza Surveillance and Response System (GISRS) regularly upload their data; in fact, participating laboratories report influenza virus detections by type, subtype, and lineage that are subsequently made available in a weekly aggregated manner. Although FluNet data are subject to local variability in testing and reporting, they undergo validation by national authorities and are regularly reviewed for quality control [5]. We stratified data by hemisphere and WHO region to account for geographic variations in seasonality and defined seasons based on geographic location: July to June for the Northern Hemisphere and January to December for the Southern Hemisphere and intertropical regions. Only complete seasons were included in the analysis; therefore, for the Northern Hemisphere, the latest analyzed season was 2023/24 (from July 2023 to June 2024), while for the Southern Hemisphere and intertropical regions, it was represented by the 2024 calendar year (from January to December). Influenza A cases were categorized as 2009 pandemic H1N1, H3N2, or “other” (including nonsubtyped and nonpandemic H1N1), while influenza B cases were classified as Victoria, Yamagata, or uncharacterized. Statistical analyses were performed at the country level (results not shown), with countries that were further grouped according to two geographical schemes: one based on the six WHO regions (Africa, Eastern Mediterranean, European, Southeast Asia, Western Pacific, and Americas) and another one based on latitudinal location (Northern and Southern Hemispheres and intertropical belt). For each country and season (country‐season), we then calculated the proportion of influenza cases attributed to each virus type (A and B). Additionally, we determined the median number of reported cases across country‐seasons and the median proportion of A detections (Median %A). A country‐season was classified as dominated by a given virus type if that type accounted for at least 50% of all reported influenza detections in that country‐season. Sensitivity analyses assessed the proportion of country‐seasons in which influenza A accounted for ≥ 80%, 50%–79%, 20%–49%, or < 20% of all reported influenza cases. Finally, the global proportion of influenza A‐dominated country‐seasons for the period was calculated, as done by Zanobini et al. [6]. Additionally, for each country‐season, we estimated the proportion of influenza cases caused by each influenza virus A subtype and B lineage. Postpandemic patterns (2022–2024) were compared with prepandemic trends and measures (2010–2020) reported in a previous study by Zanobini et al. [6].

Influenza surveillance data from 145 countries were analyzed, representing 260 influenza country‐seasons (Table 1). Globally, influenza A viruses accounted for 77.2% of all influenza detections (80.6% in 2022, 75.5% in 2023, and 74.7% in 2024) compared to 72.5% during 2010–2020 [6]. The global percentage of country‐seasons dominated by influenza A (≥ 50% of detections) increased from 84.6% between 2010 and 2020 (range: 62.6%–95.5%) to 92.3% postpandemic (range: 90.2%–97.8%) [6]. During 2022–2024, the Northern Hemisphere showed a higher proportion of influenza A cases (79.0%) than the Intertropical belt (71.9%) and the Southern Hemisphere (74.6%). Also looking at WHO regions, influenza A consistently exceeded influenza B, with medians in 2022–2024 surpassing the 2010–2020 global average in all six areas (range: 73.0%–79.5%). Table 2 shows that in 2022 A/H3N2 accounted for 51.6% of influenza A detections globally, whereas A/H1N1 comprised 17.3% (the rest was not subtyped). By 2023, A/H3N2 dropped to just under one quarter of subtyped A viruses (24.4%), while A/H1N1 rose to 40.2%, reflecting a shift in the dominance of influenza A subtype across many regions. In 2024, data show A/H3N2 returning to around 35%, with A(H1N1) at roughly 22%. For influenza B, lineage characterization indicates that B/Victoria remains the only lineage detected, making up over 15% of total cases in certain seasons, whereas influenza B/Yamagata was not detected consistently (Table 2). Importantly, efforts to subtype influenza B cases led to a decline in uncharacterized samples from 21.0% prepandemic to 7.5% in 2022–2024. Tables 3 and 4 further show regional and seasonal variations in influenza A and B proportions (Table 3) and the regional and seasonal distribution of subtypes and lineages (Table 4).

**TABLE 1:** Circulation of A and B influenza viruses according to geographical area, WHO region, and season.

Subdivision	N country‐seasons (≥ 50 cases)	N influenza cases reported to FluNet	Influenza type A		Influenza type B		Median reported cases per country‐season	Median % A	% of country‐seasons with			
			N	%	N	%			≥ 80% Influenza A	≥ 50% to 80% Influenza A	≥ 20% to 50% Influenza A	< 20% Influenza A
**Global**	260	272,464	210,437	77.2%	62,027	22.8%	400	75.8%	41.9%	50.4%	6.5%	1.2%
**Geographical area**												
*Northern hemisphere*	124	198,512	156,852	79.0%	41,660	21.0%	415	77.7%	47.6%	47.6%	4.0%	0.8%
*Intertropical belt*	122	58,295	41,900	71.9%	16,395	28.1%	345	73.0%	36.1%	53.3%	9.0%	1.6%
*Southern hemisphere*	14	15,657	11,685	74.6%	3972	25.4%	1012	76.9%	42.9%	50.0%	7.1%	0.0%
**WHO region**												
*African region*	48	21,202	15,998	75.5%	5204	24.5%	305	73.5%	35.4%	62.5%	2.1%	0.0%
*Eastern Mediterranean region*	27	25,254	18,443	73.0%	6811	27.0%	408	75.2%	33.3%	55.6%	11.1%	0.0%
*European region*	88	54,360	42,503	78.2%	11,857	21.8%	339	83.6%	53.4%	42.1%	3.4%	1.1%
*Region of the Americas*	64	137,414	106,789	77.7%	30,625	22.3%	489	72.2%	36.0%	48.4%	12.5%	3.1%
*Southeast Asia region*	16	13,207	9979	75.6%	3228	24.4%	513	75.4%	31.2%	68.8%	0.0%	0.0%
*Western Pacific region*	17	21,027	16,725	79.5%	4302	20.5%	631	75.8%	47.1%	41.2%	11.7%	0.0%
**Season**												
*2022* ^*a*^	102	108,133	87,030	80.5%	21,103	19.5%	419	74.0%	32.4%	57.8%	8.8%	1.0%
*2023* ^*b*^	113	136,216	103,395	75.2%	33,821	24.8%	363	80.9%	51.3%	40.7%	6.2%	1.8%
*2024*	45	28,115	21,012	74.7%	7103	25.3%	456	75.3%	40.0%	57.8%	2.2%	0.0%

*Note:* (a) 2022–2023 in case of Northern Hemisphere detections; (b) 2023–2024 in case of Northern Hemisphere detections.

**TABLE 2:** Proportion of influenza A subtypes and B lineages among reported cases according to geographical area, WHO region, and season.

Subdivision	A(H3N2)		A(H1N1)		A other or unsubtyped		B Yamagata		B Victoria		B uncharacterized	
	N	%	N	%	N	%	N	%	N	%	N	%
**Global**	98,985	36.3%	79,758	29.3%	31,694	11.6%	1	< 0.001%	41,769	15.3%	20,257	7.5%
**Geographical area**												
*Northern hemisphere*	74,851	37.7%	59,745	30.1%	22,256	11.2%	1	< 0.001%	27,149	13.7%	14,510	7.3%
*Intertropical belt*	20,616	35.4%	17,523	30.1%	3761	6.4%	0	0.0%	13,298	22.8%	3.097	5.3%
*Southern hemisphere*	3518	22.5%	2490	15.9%	5677	36.3%	0	0.0%	1322	8.4%	2650	16.9%
**WHO region**												
*African region*	9562	45.1%	6133	28.9%	303	1.4%	0	0.0%	4571	21.6%	633	3.0%
*Eastern Mediterranean region*	7689	30.4%	7045	27.9%	3709	14.7%	0	0.0%	2968	11.8%	3843	15.2%
*European region*	16,423	30.2%	17,745	32.7%	8335	15.3%	0	0.0%	3628	6.7%	8229	15.1%
*Region of the Americas*	49,629	36.1%	39,054	28.4%	18,106	13.2%	0	0.0%	23.756	17.3%	6869	5.0%
*Southeast Asia region*	5412	41.0%	4362	33.0%	205	1.6%	0	0.0%	2938	22.2%	290	2.2%
*Western Pacific region*	10,270	48.8%	5419	25.8%	1036	4.9%	1	< 0.001%	3908	18.6%	393	1.9%
**Season**												
*2022* ^*a*^	55,778	51.6%	18,734	17.3%	12,518	11.6%	1	< 0.001%	10,836	10.0%	10,266	9.5%
*2023* ^*b*^	33,222	24.4%	54,719	40.2%	14,454	10.6%	0	0.0%	26,106	19.1%	7715	5.7%
*2024*	9985	35.5%	6305	22.4%	4722	16.8%	0	0.0%	4827	17.2%	2276	8.1%

*Note:* (a) 2022–2023 in case of Northern Hemisphere detections; (b) 2023–2024 in case of Northern Hemisphere detections.

**TABLE 3:** Circulation of A and B influenza viruses according to geographical area, divided by season.

Geographical area and season	N country‐seasons (≥ 50 cases)	N influenza cases reported to FluNet	Influenza type A		Influenza type B		Median reported cases per country‐season	Median % A	% of country‐seasons with % A			
			N	%	N	%			≥ 80%	≥ 50% to 80%	≥ 20% to 50%	< 20%
**Northern hemisphere**												
2022–2023	61	89,217	72,821	81.6%	16,396	18.4%	498	71.5%	24.6%	67.2%	6.6%	1.6%
2023–2024	63	109,295	84,031	76.9%	25,264	23.1%	361	92.9%	69.8%	28.6%	1.6%	0.0%
**Intertropical belt**												
2022	37	13,707	10,696	78.0%	3011	22.0%	225	78.7%	46.0%	43.2%	10.8%	0.0%
2023	45	22,850	15,084	66.0%	7766	34.0%	358	69.7%	26.7%	55.6%	13.3%	4.4%
2024	40	21,738	16,120	74.2%	5618	25.8%	434	74.3%	37.5%	60.0%	2.5%	0.0%
**Southern hemisphere**												
*2022*	4	5209	3513	67.4%	1696	32.6%	1449	73.1%	25.0%	50.0%	25.0%	0.0%
*2023*	5	4071	3280	80.6%	791	19.4%	684	75.8%	40.0%	60.0%	0.0%	0.0%
*2024*	5	6377	4892	76.7%	1485	23.3%	1000	80.4%	60.0%	40.0%	0.0%	0.0%

*Note:* (a) 2022–2023 in case of Northern Hemisphere detections; (b) 2023–2024 in case of Northern Hemisphere detections.

**TABLE 4:** Circulation of influenza A subtypes and B lineages according to geographical area, divided by season.

Geographical area and season	A(H3N2)		A(H1N1)		A other or unsubtyped		B Yamagata		B Victoria		B uncharacterized	
	N	%	N	%	N	%	N	%	N	%	N	%
**Northern hemisphere**												
2022–2023	46,595	52.2%	16,331	18.3%	9895	11.1%	1	< 0.001	7837	8.8%	8558	9.6%
2023–2024	28,256	25.9%	43,414	39.7%	12,361	11.3%	0	0.0%	19,312	17.7%	5952	5.4%
**Intertropical belt**												
2022	8028	58.5%	1934	14.1%	734	5.4%	0	0.0%	2579	18.8%	432	3.2%
2023	4016	17.6%	10,257	44.9%	811	3.5%	0	0.0%	6519	28.5%	1247	5.5%
2024	8572	39.5%	5332	24.5%	2216	10.2%	0	0.0%	4200	19.3%	1418	6.5%
**Southern hemisphere**												
*2022*	1155	22.2%	469	9.0%	1889	36.3%	0	0.0%	420	8.0%	1276	24.5%
*2023*	950	23.3%	1048	25.7%	1282	31.5%	0	0.0%	275	6.8%	516	12.7%
*2024*	1413	22.1%	973	15.3%	2506	39.3%	0	0.0%	627	9.8%	858	13.5%

## 3 Conclusions

Preliminary results from our analysis of influenza global surveillance data from 145 countries during 2022–2024 indicate a ≈5% increase in the global proportion of influenza A in postpandemic seasons, an increase with potential implications for prevention strategies. This overall trend is largely driven by influenza activity in the Northern Hemisphere, which represents the majority of the countries contributing to our study. However, the proportion of country‐seasons dominated by influenza A is around or higher than 90% across all geographical areas, whereas influenza B‐dominated seasons are on the decline, compared to the 2010–2019 decade (85% dominated by influenza A) [6]. Notably, uncharacterized influenza B samples dropped from 21.0% prepandemic to 7.5% in the postpandemic period: this improvement in subtyping and surveillance capacity has enabled a more accurate assessment of B lineage dynamics, thereby supporting the argument that B/Victoria is now the only influenza B lineage circulating consistently. In intertropical regions, weaker seasonality and prolonged transmission, driven by favorable climatic conditions, lead to higher influenza B circulation than in temperate zones, as described in previous studies [7, 8].

The disappearance of influenza B/Yamagata is a major change that may have contributed to the increased dominance of influenza A and the higher prevalence of A‐driven seasons observed since 2022. This is an element of the puzzle that must be carefully considered alongside epidemiological trends, disease burden, and vaccine effectiveness data to continuously assess vaccine composition and production. In 2023, reflecting evidence that B/Yamagata no longer meaningfully circulates, WHO requested a transition to trivalent vaccines [9]. This change may offer practical advantages by shortening production times and reducing costs for the same quantity of doses, particularly benefiting low‐ and middle‐income countries, and possibly allowing WHO recommendations to be issued several weeks later each year to incorporate more recent surveillance data [10]. In the coming years, a key question will be whether the advantages afforded by a trivalent vaccine ultimately outweigh the potential benefits of rethinking a quadrivalent format: Future data on circulating influenza strains and their epidemiological impact will help clarify whether a second component is needed for broader coverage. In this regard, special attention could be given to the A(H3N2) subtype, considering its dominance, higher severity [11], and poor vaccine effectiveness in recent years [12]. Should a fourth strain become necessary, incorporating an additional A clade, and in particular an A(H3N2) clade or another emerging variant might be warranted to increase protection. Among circulating influenza viruses, H3N2 exhibits the highest rate of antigenic drift, leading to frequent vaccine mismatches and lower vaccine effectiveness. In contrast, H1N1 evolves more moderately, with stable antigenic properties and periodic resurgences, while B/Victoria remains the most genetically conserved, showing limited antigenic diversity and a more predictable seasonal pattern [13]. In the coming times, balancing the manufacturing benefits of a trivalent vaccine against the potential added protection of a quadrivalent format will also depend on these ongoing data, which must guide evidence‐based recommendations for optimal influenza prevention.

## Author Contributions

**Marco Del Riccio:** conceptualization, methodology, data curation, investigation, formal analysis, visualization, writing – original draft. **Saverio Caini:** conceptualization, writing – review and editing, validation, supervision, methodology, formal analysis, data curation. **Koos van der Velden:** conceptualization, writing – review and editing, validation, supervision. **Aura Timen:** conceptualization, validation, writing – review and editing, supervision, funding acquisition, project administration, resources.

## Conflicts of Interest

M.D.R. reports having collaborated on research projects related to the epidemiology of influenza, RSV, and SARS‐CoV‐2, funded by, or having provided consultancy services to Astrazeneca, GSK, CSL Seqirus, Moderna, and Sanofi. JvS declares that Nivel has received research grants from Sanofi, AstraZeneca, and GSK for work outside this study. The other authors report no potential conflicts of interest.

## Peer Review

The peer review history for this article is available at https://www.webofscience.com/api/gateway/wos/peer‐review/10.1111/irv.70138.

## Data Availability

The data are publicly available and can be downloaded from the WHO FluNet website.
